# Supplementary material for: HIVprotI: an integrated web based platform for prediction and design of HIV proteins inhibitors
Source: J Cheminform. 2018 Mar 9;10:12. doi: 10.1186/s13321-018-0266-y (PMC5845081; doi:10.1186/s13321-018-0266-y)
Supplement: Supplementary file 2 — Additional file 2. Source code of HIVProtI web server. [file 13321_2018_266_MOESM2_ESM.zip › HIVprotI_Source-code/hivprotI/weka-3-6-6/doc/weka/classifiers/trees/j48/InfoGainSplitCrit.html]

InfoGainSplitCrit


---


|  |  |  |
| --- | --- | --- |
| **Overview** \| **Package** \| **Class** \| **Tree** \| **Deprecated** \| **Index** \| **Help** \| **Weka's home** | |  |
| **PREV CLASS**   **NEXT CLASS** | **FRAMES**    **NO FRAMES**     **All Classes** |  |
| SUMMARY: NESTED \| FIELD \| CONSTR \| METHOD | DETAIL: FIELD \| CONSTR \| METHOD |  |


---


## weka.classifiers.trees.j48 Class InfoGainSplitCrit

```
java.lang.Object
  weka.classifiers.trees.j48.SplitCriterion
      weka.classifiers.trees.j48.EntropyBasedSplitCrit
          weka.classifiers.trees.j48.InfoGainSplitCrit
```

**All Implemented Interfaces:**: java.io.Serializable, RevisionHandler

---

``` public final class InfoGainSplitCrit extends EntropyBasedSplitCrit ```

Class for computing the information gain for a given distribution.

**Version:**
:   $Revision: 1.10 $

**Author:**
:   Eibe Frank (eibe@cs.waikato.ac.nz)

**See Also:**: Serialized Form

---

**Constructor Summary**

`InfoGainSplitCrit()`


| **Method Summary** | |
| --- | --- |
| `java.lang.String` | `getRevision()`             Returns the revision string. |
| `double` | `splitCritValue(Distribution bags)`             This method is a straightforward implementation of the information gain criterion for the given distribution. |
| `double` | `splitCritValue(Distribution bags, double totalNoInst)`             This method computes the information gain in the same way C4.5 does. |
| `double` | `splitCritValue(Distribution bags, double totalNoInst, double oldEnt)`             This method computes the information gain in the same way C4.5 does. |

**Methods inherited from class weka.classifiers.trees.j48.EntropyBasedSplitCrit**

`logFunc, newEnt, oldEnt, splitEnt`

**Methods inherited from class weka.classifiers.trees.j48.SplitCriterion**

`splitCritValue, splitCritValue, splitCritValue`

**Methods inherited from class java.lang.Object**

`equals, getClass, hashCode, notify, notifyAll, toString, wait, wait, wait`

**Constructor Detail**

### InfoGainSplitCrit

```
public InfoGainSplitCrit()
```


**Method Detail**

### splitCritValue

```
public final double splitCritValue(Distribution bags)
```

:   This method is a straightforward implementation of the information
    gain criterion for the given distribution.

    :   **Overrides:**: `splitCritValue` in class `SplitCriterion`
    :   **Returns:**: value of splitting criterion. 0 by default

---


### splitCritValue

```
public final double splitCritValue(Distribution bags,
                                   double totalNoInst)
```

:   This method computes the information gain in the same way
    C4.5 does.

    :   **Parameters:**: `bags` - the distribution: `totalNoInst` - weight of ALL instances (including the ones with missing values).

---


### splitCritValue

```
public final double splitCritValue(Distribution bags,
                                   double totalNoInst,
                                   double oldEnt)
```

:   This method computes the information gain in the same way
    C4.5 does.

    :   **Parameters:**: `bags` - the distribution: `totalNoInst` - weight of ALL instances: `oldEnt` - entropy with respect to "no-split"-model.

---


### getRevision

```
public java.lang.String getRevision()
```

:   Returns the revision string.

    :   **Returns:**: the revision


---


|  |  |  |
| --- | --- | --- |
| **Overview** \| **Package** \| **Class** \| **Tree** \| **Deprecated** \| **Index** \| **Help** \| **Weka's home** | |  |
| **PREV CLASS**   **NEXT CLASS** | **FRAMES**    **NO FRAMES**     **All Classes** |  |
| SUMMARY: NESTED \| FIELD \| CONSTR \| METHOD | DETAIL: FIELD \| CONSTR \| METHOD |  |


---
